# The Evaluation of Cases with Y-Chromosome Gonadal Dysgenesis: Clinical Experience over 18 Years

**DOI:** 10.4274/jcrpe.4826

**Published:** 2018-02-26

**Authors:** Merih Berberoğlu, Zeynep Şıklar, Ankara University Faculty of Medicine Disorders of Sexual Development Ethics Committee

**Affiliations:** 1 Ankara University Faculty of Medicine, Department of Pediatric Endocrinology, Ankara, Turkey; 2 Includes all members from 1996 to 2017

**Keywords:** Gonadal dysgnesis, 46, XY, 45, X/46, XY

## Abstract

**Objective:**

Y-chromosome gonadal dysgenesis (GD) is a rare subgroup of disorders of sexual development (DSD) which results from underdeveloped testis and may exhibit heterogenous symptoms. These patients are phenotypically classified into two groups - complete and partial, and their karyotypic description is either 46,XY GD or 45,X/46,XY GD. In this study; we aimed to evaluate the characteristics of cases with Y-chromosome GD.

**Methods:**

Thirty eight cases were followed-up between 1998 and 2016. The age of admission ranged between 0 and 17 years. Clinical and laboratory findings as well as follow-up characteristics of the cases were evaluated retrospectively from the patient files.

**Results:**

There were 26 cases (four complete, 22 partial) in the 46,XY GD group, and 12 cases (four complete, 8 patients with complete GD in the 45,X/46,XY. Mean age at admission was 6.2±4.6 years for all cases. Patients with complete GD in the 45,X/46,XY GD group were diagnosed earlier that the patients with complete GD in the 46,XY group [11 years vs. 14.31 years of age (p<0.01)]. There were no additional findings in 55% of all patients. In the remaining 45% additional clinical findings, mainly short stature, were detected in 75% of the patients in the 45,X/46,XY GD and 30% of the patients in the 46,XY GD groups. All patients with complete 46,XY and 45,X/46,XY GD were raised as females. There was no gender dysphoria in patients that were raised as females, except for one case. Gonadectomy was performed in 14 patients, at a mean age of 8.75±2.3 years and pathological assessment of the gonads was reported as normal in all cases.

**Conclusion:**

Y-chromosome GD is a very heterogenous clinical and genetic disorder and requires a multifaceted approach to management. Whether including syndromic features or not, associated clinical features may lead to earlier diagnosis, especially in complete forms of GD. Due to difficulties encountered in the long-term follow-up of these patients, evaluation of appropriateness of sex of rearing decision is not truly possible. Performance of gonadectomy during the first decade appears be a preventive factor for tumor development since these tumors are usually seen during the second decade.

## What is already known on this topic?

Gonadal dysgenesis is rare and is the most complicated subgroup of disorders of sexual development. It results from underdeveloped gonads. Small case series have been published.

## 

### What this study adds?

Characteristics of Y-chromosome gonadal dysgenesis are presented in a large group. Specific characteristics of patients that provided important clues for follow-up are discussed.

## Introduction

Gonadal dysgenesis (GD) is a rare condition and is the most complicated subgroup of disorders of sexual development (DSD) which result from underdeveloped gonads. DSDs are phenotypically classified into two groups, as complete and partial; while their karyotypic description is 46,XY GD and 45,X/46,XY GD which may both occur in either group (1,2,3). The term “Y-chromosome GD” is used for both 46,XY and 45,X/46,XY GD. Histologically 45,X/46,XY individuals can have bilateral streak gonads, bilateral dysgenetic gonads or a unilateral streak and contralateral dysgenetic gonad. This latter form is termed as mixed 45,X/46,XY GD (4,5). Complete Y-chromosome GD is characterized by female external genitalia, bilateral streak gonads and hypergonadotropic hypogonadism in 46,XY GD and 45,X/46,XY GD patients. Absence of anti-Müllerian hormone (AMH) leads to a normally developed Müllerian duct. Patients with partial Y-chromosome GD may present with variable degrees of impaired testicular development and testicular function. Phenotypic appearance is related to the level of functional testicular hormones (6). The amount of AMH production also determines the degree of regression of Müllerian structures. Patients with partial GD have bilateral dysgenetic testis or a unilateral dysgenetic testis and contralateral streak gonad with ambiguous genitalia (7). Sex chromosome abnormalities or mutation in genes of transcription factors which are required for normal development of gonads may lead to Y-chromosome GD. Several transcriptional factors such as SRY, SOX9, NR5A1, MAP3K1, GATA4, FOG2, DHH, CBX2 and ATRX all have roles in testicular development (8). Additional systemic findings are frequently seen in patients with Y-chromosome GD. It is accepted that 46,XY GD and 45,X/46,XY DSD with defective testis development or function are associated with the greatest risk of neoplasia (9). Thus there is extra concern about patients who have the increased risk of germ cell neoplasms in the dysgenetic gonads (7,10,11,12). Early and correct diagnosis of Y-chromosome GD has important clinical implications, not only for gonadal intervention because of the higher potential malignancy risk and for the timing of gonadectomy, but also for sex of rearing. In this study we aimed to evaluate the characteristics of the cases with GD followed-up in our clinic and provide the benefit of our long-standing experience which may be helpful in the diagnosis and treatment of these patients.

## Methods

In this retrospective study, patients with Y-chromosome GD admitted to the pediatric endocrinology clinic between June 1998 and December 2016 were evaluated. Patients with Turner syndrome, Klinefelter syndrome, Ovotesticular syndrome and 46,XX GD were excluded from the study. Of patients with gonadal developmental disorders, nine had been diagnosed as ovotesticular syndrome and five as 46,XX GD. Patients with Y-chromosome GD consisted of 38 children and age at presentation ranged from newborn to 17 years old. Clinical and laboratory findings including the patients’ phenotypic appearance, karyotypes, imaging of gonads and internal genital structures by ultrasonography and/or magnetic resonance imaging, histopathological evaluation of gonads, functioning of gonads, sex of rearing and additional systemic findings were evaluated. The diagnosis of complete Y-chromosome GD was considered in cases with bilateral streak gonads and female internal and external genitalia. The diagnosis of partial Y-chromosome GD was made by the findings of 46,XY or 45,X/46,XY karyotype, ambiguous genitalia, low testeosterone response to human chorionic gonadotropin (hCG) stimulation test at prepubertal ages, low basal testosterone levels at pubertal ages, low AMH levels, bilaterally or unilaterally dysgenetic gonads and presence of a contralateral streak gonad (13). External genitalia was rated using the Sinnecker classification (14). Laparotomy was performed as required. Pathological studies were obtained by laparotomy and/or gonadectomy and conducted in 20 patients. Histopathological examination of gonads was carried out either on resected or biopsied tissue. Chromosomal analysis was done by evaluating metaphase G bands prepared from peripheral blood lymphocytes. When required, specific molecular analysis [fluorescent *in situ* hybridization, polymerase chain reaction (PCR), gonadal tissue sytology] was performed. Patients with Y-chromosome GD were divided into 46,XY GD and 45,X/46,XY GD karyotypically. Hormonal assessment in serum samples was performed using the immuno-chemiluminescence method for luteinising hormone, follicle-stimulating hormone (FSH) and serum total testosterone levels on the Advia Centaur XP^®^, Siemens Healtcare GmbH, Germany. AMH concentrations were assessed using an enzyme-linked immunosorbent assay on the Access 2 Immunassay Analyser^®^, Beckman Coulter, Inc, California, United States. For determination of Leydig cell function, basal and hCG stimulated testosterone levels were determined when required. The hCG stimulation protocol was administration of three intramuscular injection of hCG on successive days in an age dependent daily dose (age <1 year, 500 units; 1-10 years, 1000 units; >10 years, 1500 units) (15). Gender assignment was approved by the DSD Ethics Committee of the University. DSD Ethics Commitee members consisted of a pediatric endocrinologist, an adult endocrinologist, a plastic surgeon, a pediatric surgeon, a medical geneticist, a child psychiatrist, a pediatric urologist and a medical ethics specialist. Follow-up characteristics of the patients such as appropriateness of sex of rearing by psychiatric evaluation, development of gonadal tumors or additional problems (short stature, cardiac problems, renal abnormalities etc.) were evaluated. Frequencies and percentages represented the descriptive statistics for categorical variables and mean ± standard deviation values were used for continuous variables by using SPSS for Windows v. 22.0 statistical software. Student’s t-test were used for parametric variables between groups. p<0.05 was accepted as significant. Inform consent were given by parents after evaluation by DSD Committee. Ethical approved was given by the Ankara University Ethical Comittee for Clinical Research (approval number: 15-639-15).

## Results

### Admission Characteristics

**Figure 1:**
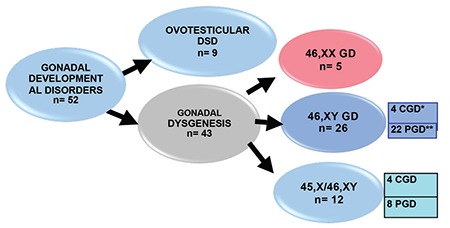
The distribution of patients with gonadal developmental disorders *CGD: complete gonadal dysgenesis, **PGD: partial gonadal dysgenesis, DSD: disorders of sexual development, GD: gonadal dysgenesis

During the study period, 38 cases with a mean age of presentation of 6.2±4.6 years, were diagnosed as Y-chromosome GD. There were 26 cases of 46,XY GD and 12 cases of 45,X/46,XY GD. At presentation, the mean ages were 6.72±5.2 years for 46,XY GD and 5.14±4.1 years for the 45,X/46,XY GD patients (Table 1 and Figure 1). In our cohort, the number of patients with 46,XY GD was nearly double of the patients with 45,X/46,XY GD (Figure 1). In the 46,XY GD group, four patients phenotypically had complete GD, 22 patients had partial GD and presented with genital ambiguity. Sinnecker scores of patients were from 2 to 5. Müllerian structures were persistent in 12 of 46,XY GD patients. Gonads were bilaterally streak gonad in six patients, bilaterally dysgenetic testis in 14 patients. Two patients were of 46,XY karyotype and phenotypically one was partial GD and the other was complete GD. This latter case had embryonic testicular regression syndrome (ETRS). *SRY* gene deletion was not detected in any of the cases. Precise genetic diagnosis could be made in three patients; two with SF1 and one with WT1 mutations. In the patients with SF1 mutation diagnosis had been made at the age of ten years in the first and in the neonatal period in the second patient. The older of these patients had been raised as female and admitted to our clinic with virilisation of external genitalia. Hormonal analysis showed high FSH and high testosterone levels. The gonads were bilateral dysgenetic testes and were located in the abdomen. The Müllerian duct had not regressed completely. The other SF1 mutated patient had Sinnecker 2a external genitalia with bilaterally inguinal testis and no Müllerian duct. He was raised as male without any health problems. The patient with a mutation of the *WT1* gene was diagnosed as Denys-Drash syndrome. Phenotypically he was partial GD. During follow-up his nephropathy progressed to end stage renal disease. Although not genetically proven, one patient within the 46,XY group probably had SOX9 mutation. He had partial GD and skeletal abnormalities including craniosynostosis and camptodactyly. A further patient with 46,XY partial GD had lissencephaly with absence of corpus callosum, intractable seizures, and choroid coloboma suggesting the aristaless-related homeobox (*ARX*) gene mutation. Unfortunately this patient died in the neonatal period and no genetic studies could be undertaken. In the 45,X/46,XY GD group, phenotypically four had complete GD and the remaining eight patients had partial GD. Of the 45,X/46,XY patients, four had unilateral dysgenetic testis and contralateral streak gonad, three had bilaterally streak gonads and the rest had bilaterally dysgenetic testis. All cases with 45,X/46,XY GD had Müllerian ducts exhibiting varying degrees of regression. Patients with complete GD in the 45,X/46,XY GD group were diagnosed at a younger age than the patients with complete GD in the 46,XY group (11 years vs. 14.31 years respectively, p<0.01). There were no additional findings in 55% of all patients. Among the syndromic cases, additional clinical findings were detected in 75% in the 45,X/46,XY GD (9/12) and 31% in the 46,XY GD (8/26) groups. Short stature was the most frequently encountered additional finding and was detected in six patients with 45,X/46,XY GD and in two patients with 46,XY GD (Table 2). Mean duration of follow-up was 7.3±3.8 years. Sex assignment was male in 24 patients and female in 12 patients (Table 3). Decision of sex assignement was not made in only one 46,XY infant admitted to our clinic at an age of seven months with Sinnecker 3 external genitalia. The last evaluation of this patient was at age 1.7 years and at this time the patient had been given a female name by the family and psychologic evaluation is continuing.

### Follow-up Characteristics

Long term follow-up of patients was complicated by poor attendance in some patients for a number of reasons such as financial problems, families giving more importance to additional systemic findings than the genital problems, transfer to another clinic, etc. One case with severe congenital abnormalities died in the neonatal period. 

Thirteen patients were raised as females, seven in the 46,XY GD group and six in the 45,X/46,XY GD group. Psychiatric evaluation is ongoing in one infant and the sex assignment has not yet been made. There was no gender dysphoria in patients that were raised as females, except one with 45,X/46,XY GD. This patient had been diagnosed at age 1.5 years and sex assignment was made as female at first evaluation. Female correction surgeries were undertaken. Unfortunately the patient did not reattend clinic until 14 years of age, at which time he had reassigned himself as a male. The patient was reevaluated psychologically and the local ethic committee decided that he had a male gender identity. Apart from this patient, 19 patients (six in the 45,X/46,XY GD and 13 in the 46,XY GD group) who had attained pubertal ages showed no gender dysphoria. 

Gonadectomy was carried out in 14 of these patients. Mean age of this group was 8.75±2.3 years. Orchidopexy was performed in eight patients. Gonadal pathology results were normal in all cases and showed no malignancy.

## Discussion

Y-chromosome GD shows a wide spectrum of phenotypic, genetic and histopathological characteristics and constitutes a heterogenous group within gonadal developmental disorders. Among the etiological factors, there are a range of genetic abnormalities. During testis development, several genes are activated and expressed at different times (1). Due to this genetic heterogenity in etiology, it is not suprising to observe extremely heterogenous clinical findings in these patients. Despite extensive analysis, no definite etiology can be established in an important proportion of gonadal developmental disorders (16). In our study sample, genetic etiology of cases with Y-chromosome GD could only be established in three patients. Two of these patients had SF1 mutations and the other had WT1 mutation. In patients with 45X/46,XY GD, no additional genetic studies were needed. In the 46,XY GD group, *SRY* gene deletion was not detected in any of the cases. Because analysis of mutations in the *SF1* gene has become available only recently for our patients, this analysis could not be performed in any of the patients. WT1 mutation could be analyzed only in one patient with Denys-Drash syndrome clinically. Future advances in genetic analysis will be helpful in exploring etiology further. SF1 mutation was detected in two patients who showed clinically distinctive features. It is known that the clinical spectrum of the *SF1* gene mutation is very heterogeneous and that it causes either a female phenotype or an ambiguous genital structure in 46,XY individuals. It has been reported that in a small number of cases with SF1 gene mutation, virilization can be observed in puberty (17,18). Although we have only two patients in this series with SF1 mutation, they demonstrate how this disorder can exhibit clinical heterogeneity with one presenting at a young age and the other presenting in late childhood with virilization. These cases also illustrate that clinical heterogenity of Y-chromosome GD patients depends not only on genetically different gene mutations, but also on a variety of presentations in patients with the same mutation. Syndromic Y-chromosome GD was found in two patients (Denys-Drash and Camptomelic Dysplasia syndromes). These patients presented with severe extragenital systemic findings; genital problems may be overlooked in such cases. Thus, genital abnormalities should be given more attention in cases with syndromic features. Low testosterone and low AMH levels also may be a clue in GD patients who have syndromic features.

In our study group, 55% of patients had no additional systemic findings. The presence of any systemic feature, apart from genital abnormalities, may actually be helpful in leading to an early diagnosis in these patients. Complete GD without additional abnormalities may only be diagnosed at pubertal ages due to a delay in pubertal signs (19). In our series, additional findings such as short stature, webbed neck and coarctation of the aorta were more frequent in the 45,X/46,XY GD group than in the 46,XY GD. Short stature was the most frequently detected finding (Table 2). It is well known that stigmata of Turner syndrome are common in children with 45,X/46,XY mosaicism. Monogenic X-chromosome is blamed for most of these features (20,21). In the case of 45,X/46,XY GD, there is a simultaneous presence of a lineage with X monosomy and XY cell lines among the tissues. The different tissue distributions of the 45,X and 46,XY chromosomal cell lines presumably reflect the wide variety of phenotypes observed (22). 45,X/46,XY mosaicism may be seen in cases with completely normal male external genitalia and it has been suggested that this phenotype is the most common among patients with 45,X/46,XY mosaicism (22). Patients with complete GD may only present because of pubertal delay. If there are additional phenotypic features, this may lead to earlier diagnosis, as was true in our series. While three patients with complete 45,X/46,XY GD were diagnosed before 10 years of age, all cases with complete 46,XY GD came to medical attention due to pubertal delay after the age of 14 years. Within the 46,XY GD group, two patients had been diagnosed as ETRS. ETRS has been considered to be a part of the clinical spectrum of partial 46,XY GD (23). The time of testicular regression (early or late) determines the degree of virilization of the external genitalia and regression of the Müllerian duct. Long term follow-up of GD patients involves many difficulties. Precise decision making on sex of rearing, time of gonadectomy and time of corrective surgery can be taxing. Associated clinical features may be present in these patients which will also require investigation and treatment. Sex of rearing may be the most important issue in patients with GD. In fact, in patients with complete GD, the sex of rearing decision is usually female. Controversies occur mostly for patients with partial GD (5). In our study, sex assignment was made in all patients with complete GD, and no gender dysphoria was seen. In the partial GD group, only one case showed gender dysphoria. Although most of our cases seem to be successfully adapted to their selected gender, difficulties in long term follow-up of cases prevent us from definitively reporting on this issue. Despite a mean duration of follow-up in our cohort of 7.3±3.8 years, a substantial proportion of followed-up patients are still at prepubertal ages. Only 19 of the 38 patients attained puberty. At this stage, we are not able to make a complete evaluation for appropriateness of sex of rearing in this group. It is important to avoid performing radical surgery in patients before they are given their precise sex. It should always be remembered that every GD patient is unique and has to be treated with individualized care and with a multidisciplinary approach (24). Gonadal tumor development is one of the most important challenges in patients with GD. Gonadal tumor risk is highest in 46,XY GD 46,XY DSD (11). The most common tumor observed in complete and/or partial GD is gonadoblastoma (12). The timing of gonadectomy before tumor development is a very important issue in patients with GD (10,11,12). In our series, gonadectomy was performed during the first decade and this seems to be an acceptable regimen for prevention of tumor development, since these tumors are usually seen during the second decade. Risk of gonadoblastoma is high when the early stage of Sertoli cell differentiation is disrupted by mutations in SRY, WT1, SOX9, DMRT1, FOG2/GATA4, FGF9 etc. (8,25). Patients with 45,X/46,XY GD also have disturbed early Sertoli cell development. The presence of gonadoblastoma Y locus is also a prerequiste for development of malignancy. Neoplastic transformation of germ cells in dysgenetic gonads (gonadoblastomas and/or an invasive germ cell tumor) occurs in 20-30% of 46,XY DSD patients (25). Thus, careful evaluation of gonads by imaging and/or histology is critical. The risk of germ cell tumor increases with age and undescended testis is an additional risk factor (11). In complete 46,XY GD patients, it is suggested that bilateral gonadectomy should be performed before pubertal age to avoid degeneration of dysgenetic tissue (5). In partial 46,XY GD, there is inconsistency in opinion with respect to timing of gonadectomy. A more individualized and conservative approach in the decision-making process for gonadectomy, by taking into account certain factors including location of the gonads, internal and external phenotype and sex of rearing, has being emphasized in recently (5). Provided the gonad is functional and easily accessible to palpation and imaging studies, some authors propose that gonadectomy can be postponed and imaging should be performed annually (25). Bilateral gonadectomy was recommended in patients with XY partial GD with nonscrotal gonads that cannot be surgically repositioned into the scrotum (5).

### Study Limitations

Limitations of our study are irregular clinic attendance of patients. 

## Conclusion

Y-chromosome GD is an extremely heterogenous clinical and genetic disorder with variations in diagnosis, treatment and also in approach to additional problems. Each patient should be evaluated individually using a multidiciplinary approach. Whether the patient has syndromic features or not, associated clinical features can lead to earlier diagnosis, especially in complete forms of GD. Difficulties in long-term follow-up constitute an obstacle to judging the appropriateness of the sex of rearing decision. Gonadectomy during the first decade seems to be protective against tumor development which usually occurs during the second decade in these patients. 

## Figures and Tables

**Table 1:**
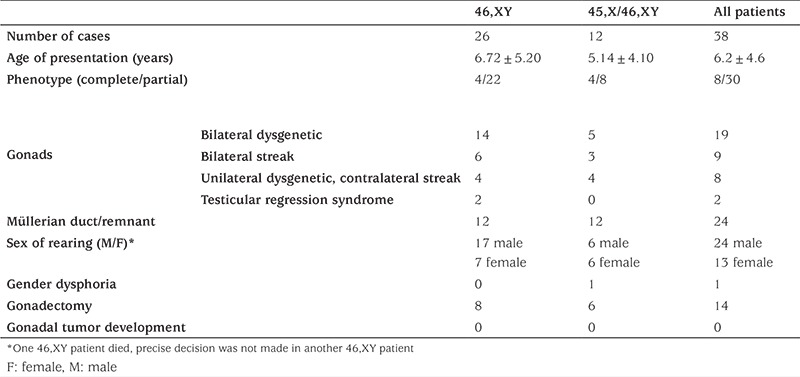
Characteristics of patients

**Table 2:**
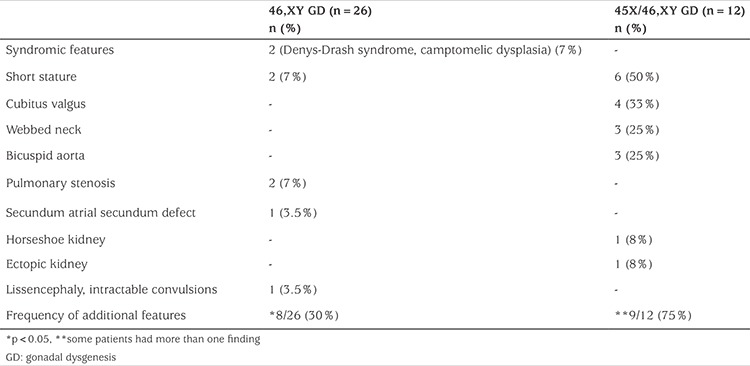
Additional clinical features of Y-chromosome gonadal dysgenesis patients

**Table 3:**
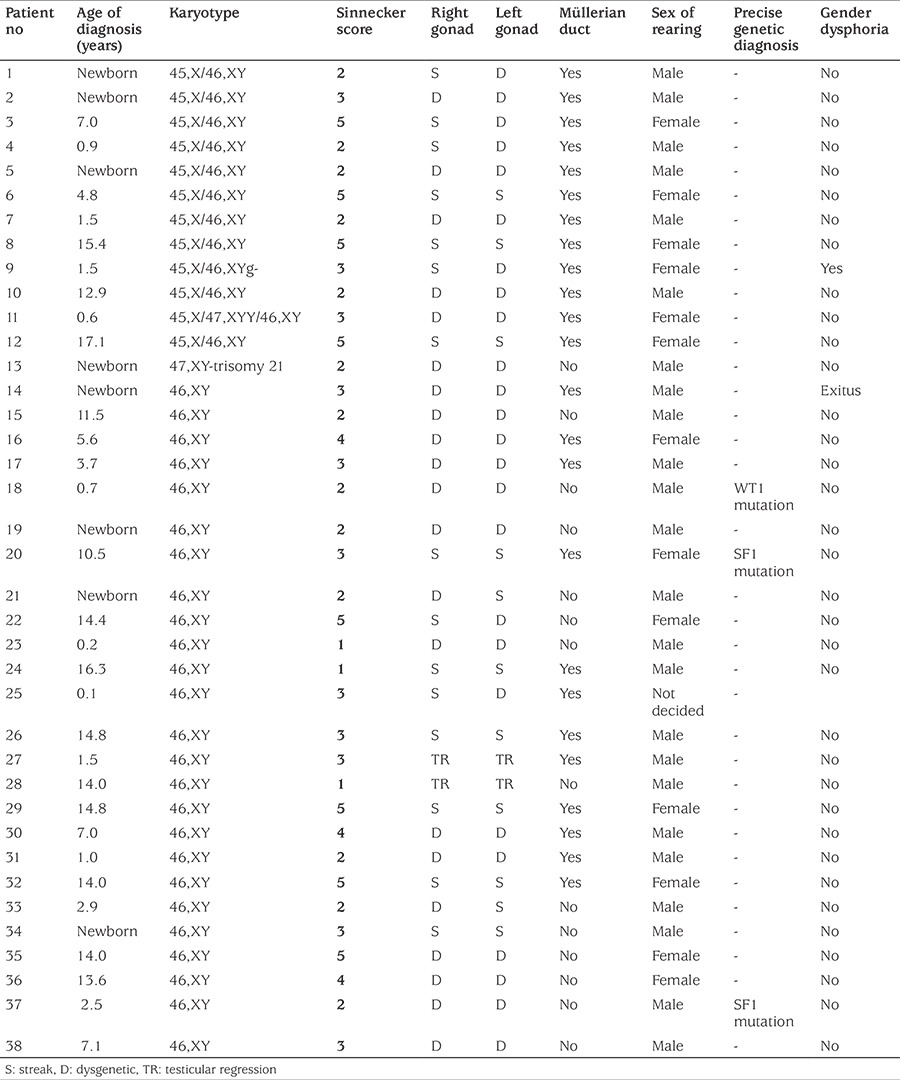
Phenotypic characteristics and sex of rearing in all patients
